# A Cyclic Peptide Based on Pheasant Cathelicidin Inhibits Influenza A H1N1 Virus Infection

**DOI:** 10.3390/antibiotics13070606

**Published:** 2024-06-28

**Authors:** Yaping Pei, Zhihua Chen, Ruihan Zhao, Yanxing An, Haiche Yisihaer, Chaojie Wang, Yaning Bai, Libin Liang, Lin Jin, Yongting Hu

**Affiliations:** Shanxi Key Laboratory for Modernization of TCVM, College of Veterinary Medicine, Shanxi Agricultural University, Taigu 030801, China; b20211058@stu.sxau.edu.cn (Y.P.); s20212417@stu.sxau.edu.cn (Z.C.); z20213702@stu.sxau.edu.cn (R.Z.); z20213683@stu.sxau.edu.cn (Y.A.); s20222385@stu.sxau.edu.cn (H.Y.); z20223811@stu.sxau.edu.cn (C.W.); s20212398@stu.sxau.edu.cn (Y.B.); lianglibin@sxau.edu.cn (L.L.)

**Keywords:** antiviral peptide, influenza A virus, type I interferon, cyclic peptide

## Abstract

Influenza viruses are the leading cause of upper respiratory tract infections, leading to several global pandemics and threats to public health. Due to the continuous mutation of influenza A viruses, there is a constant need for the development of novel antiviral therapeutics. Recently, natural antimicrobial peptides have provided an opportunity for the discovery of anti-influenza molecules. Here, we designed several peptides based on pheasant cathelicidin and tested their antiviral activities and mechanisms against the H1N1 virus. Of note, the designed peptides Pc-4 and Pc-5 were found to inhibit replication of the H1N1 virus with an IC50 = 8.14 ± 3.94 µM and 2.47 ± 1.95 µM, respectively. In addition, the cyclic peptide Pc-5 was found to induce type I interferons and the expression of interferon-induced genes. An animal study showed that the cyclic peptide Pc-5 effectively inhibited H1N1 virus infection in a mouse model. Taken together, our work reveals a strategy for designing cyclic peptides and provides novel molecules with therapeutic potential against influenza A virus infection.

## 1. Introduction

The influenza virus is the primary pathogen causing seasonal influenza globally. Data from the World Health Organization (WHO) indicate that each year, influenza epidemics lead to approximately 3 to 5 million severe infections and result in an estimated 200,000 to 600,000 deaths (https://www.who.int/health-topics/influenza-seasonal, accessed on 1 June 2024). Influenza viruses are categorized into types A, B, C, and D [1]. Among them, type A influenza is most prevalent during flu season, causing a spectrum of illnesses ranging from mild to severe, and it can infect both humans and animals [2]. Currently, vaccination is the main preventive measure; however, antigenic drift may make the currently prevalent viral strains different from the vaccine strains. This divergence can reduce vaccine efficacy, particularly in vulnerable populations such as elderly individuals and individuals of all ages with immune-compromising diseases [3].

Antiviral medications represent a crucial component of pandemic preparedness, particularly in the initial phases when strain-specific vaccines are not yet available. To date, only four drugs have received regulatory approval and are recommended for both chemoprophylaxis and therapeutic intervention against influenza in different regions [4]. Three of them (oseltamivir, zanamivir, peramivir) are neuraminidase inhibitors (NAIs) that prevent viral release from infected cells [5]. Baloxavir marboxil is a newly approved antiviral against influenza via the inhibition of cap-dependent endonuclease (CEN) [6]. The advent of these drugs has significantly reduced the illness duration, mitigated disease severity, and substantially contributed to curbing influenza transmission and outbreaks. Nonetheless, concerns regarding their side effects and the emergence of drug resistance have limited their clinical application [7,8,9]. Consequently, there is an imperative to develop innovative, safe, and efficacious antiviral drugs to combat influenza viruses effectively.

Over the past several decades, there has been an emphasis on identifying novel synthetic and natural compounds that exhibit beneficial antiviral properties. Within this new generation of compounds, antiviral peptides have emerged as a distinct class of small biomolecules. Typically characterized by molecular weights between 2000 and 10,000 daltons, these peptides have demonstrated an exceptional antiviral potential. This is attributed to their capacity to penetrate cellular membranes and viral envelopes with relative ease [10]. The antiviral peptides directly bind to a viral envelope or capsid proteins, disrupting the viral structure and thus inhibiting viral infection and replication. Furthermore, the antiviral peptides can impede the viral life cycle at various stages, including the adsorption, fusion, endocytosis, and release of viruses from host cells. Notably, some of the peptides possess the ability to modulate the host immune response, thereby bolstering the body’s innate defense mechanisms against viral invasion [11,12]. Due to their diverse mechanisms of action and susceptibility to degradation by peptidases in vivo, antiviral peptides have the potential to provide broad-spectrum antiviral effects while simultaneously reducing the risk of cumulative and long-term side effects, offering a promising avenue for antiviral therapy. Additionally, many antiviral peptides exhibit low toxicity to host cells, which contributes to a favorable safety profile suitable for clinical applications. These peptides can be sourced from a variety of natural sources, including plants, animals, and microorganisms, or they may be derived from the human immune system itself. Moreover, advancements in biotechnology have allowed for the artificial design and synthesis of peptides tailored to specific antiviral applications [13].

Hosts, including humans, insects, and wild birds, serve as critical reservoirs for the discovery of peptide drugs with potential therapeutic efficacy. Among wild avian species, pheasant (*Phasianus colchicus*) is recognized as a natural host for influenza A viruses, which are capable of continuously carrying and transmitting multiple influenza virus subtypes [14]. These strains cause only mild or subclinical symptoms in wild birds [15,16]. This phenomenon implies that pheasants have likely developed an array of physiological and behavioral adaptations to bolster their resistance to diseases within their intricate natural habitat. These adaptations may be attributed to the presence of an immune system that is capable of swiftly identifying and combating pathogen incursions. Additionally, it is plausible that pheasants possess endogenous molecules with inherent antiviral properties, which contribute to their enhanced resilience against viral infections. Cathelicidins are a class of host defense peptides found in vertebrates that typically contain a highly conserved N-terminal cathelin structural domain and a highly variable C-terminal mature peptide region. The mature peptide region is a major part of their biological activity [17]. Extensive research has demonstrated that cathelicidins possess not only antimicrobial properties but also a spectrum of immune-modulatory functions [18,19,20,21]. In addition, the potential of cathelicidins in defending against viral infections has received extensive attention from researchers. For example, LL-37, a host defense peptide, can reduce Human Rhinovirus 1B (HRV1B) replication in airway epithelial cells [22]. WL-1, an antiviral peptide derived from human cathelicidin, demonstrated a potent inhibitory effect on HSV-1 [23]. Hg-CATH and Pb-CATH4 are cathelicidins from *Heterocephalus glaber* and *Python bivittatus* that significantly reduced HSV-1 DNA replication and the production of infectious viral particles in keratinocytes at noncytotoxic concentrations, with stronger activity of Pb-CATH4 [24]. Chicken-derived CATH-B1 showed favorable antiviral activity against avian influenza virus (IAV) by binding to the viral particle and thereby blocking viral entry [25]. The antiviral potential of chicken CATH-2 in vivo against infectious bronchitis virus (IBV) infection also showed positive prospects [26]. While studies have established the antimicrobial function of pheasant cathelicidins, there are no studies on the effects of these peptides on viral infection [27].

In this study, we investigated the antiviral activity and underlying mechanisms of pheasant cathelicidin and its derived peptides against influenza A virus. Our findings may offer an important strategy for the development of novel antiviral drugs.

## 2. Results

### 2.1. Design of Cathelicidin Pc-1 and Its Derivative Peptides

**Figure 1:**
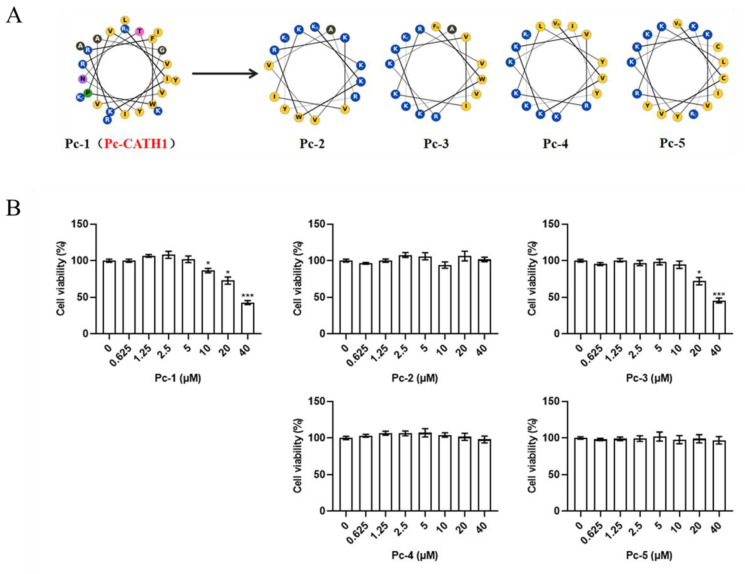
The amphipathic helix structure and cytotoxicity of cathelicidin Pc and its derivative peptides. (**A**) The Schiffer–Edmundson wheel representation of the designed peptides. (**B**) A549 cells were treated individually with five peptides for 48 h and cell viability was measured by a CCK-8 assay. The data represent 3 independent experiments and are presented as the mean ± SEM. Statistical significance was determined by a two-tailed Student’s *t*-test. * *p* < 0.05; *** *p* < 0.001.

To identify a template for antiviral peptides, we conducted a search in the Antimicrobial Peptide Database (APD) and identified the cathelicidin sequence of *Phasianus colchicus*. This sequence was designated Pc-1. Subsequently, we optimized its sequence to obtain derivative peptides, named Pc-2, Pc-3, Pc-4, and Pc-5 (Figure 1A). The safety of the designed peptides on A549 cells was assessed using the CCK-8 assay. Within the tested concentrations, Pc-1 exhibited the most constrained working concentration range, showing cytotoxicity at concentrations above 10 µM. After optimization, the cytotoxicity of Pc-3 decreased. Furthermore, all doses of Pc-2, Pc-4, and Pc-5 were found to be nontoxic (Figure 1B).

### 2.2. The Cathelicidin Pc-Derived Peptides 4 and 5 Exhibited the Strongest Inhibitory Effects on H1N1 Virus Infection In Vitro

**Figure 2:**
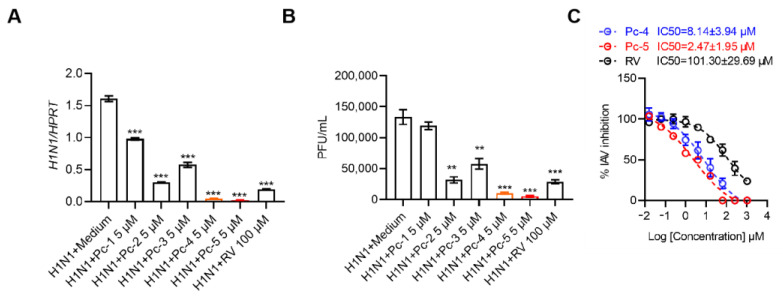
Cathelicidin Pc-1 and its derivative peptides exhibit antiviral activity against H1N1 in vitro. (**A**) qPCR analysis of H1N1 in A549 cells after 24 h of infection with H1N1 (MOI = 0.01) alone or in combination with 5 µM cathelicidin Pc-1, Pc-2, Pc-3, Pc-4, Pc-5 or 50 µM RV. The results are presented relative to the reference gene HPRT and H1N1 + medium groups. (**B**) H1N1 titration in the supernatants of A549 cells after infection with H1N1 (MOI = 0.01) alone or in combination with 5 µM cathelicidin Pc-1, Pc-2, Pc-3, Pc-4, Pc-5 and 50 µM RV. (**C**) The IC50s of Pc-4 and Pc-5 against H1N1 infection were detected. In addition, RV was selected as a positive control. The data represent 3 independent experiments and are presented as the mean ± SEM. Statistical significance was determined by a two-tailed Student’s *t*-test. ** *p* < 0.01; *** *p* < 0.001.

We next performed a qRT-PCR to assess the antiviral activity of the designed peptides against the H1N1 virus at 24 h post-infection. Encouragingly, all five peptides significantly inhibited the H1N1 virus infection in A549 cells, with the peptides Pc-4 and Pc-5 inducing the most robust antiviral response (Figure 2A). Consistent with previous results, a plaque formation assay also indicated that the designed peptides significantly reduced the production of viral particles, especially Pc-4 and Pc-5 (Figure 2B). The 50% inhibitory concentration (IC50) is a pivotal parameter for evaluating the efficacy of antiviral drugs. We meticulously conducted experiments to determine the IC50 values for the peptides Pc-4 and Pc-4 utilizing ribavirin (RV) as a positive control. Notably, the IC50 value for Pc-4 was determined to be 8.14 ± 3.94 µM, while Pc-5 exhibited even more pronounced antiviral activity, with an IC50 value of 2.47 ± 1.95 µM. In contrast, the performance of RV was relatively inferior, with an IC50 value over tenfold greater, reaching 101.30 ± 29.69 µM (Figure 2C).

### 2.3. The Cyclic Peptide Pc-5 Induces Type I Interferon and Downstream Gene Expression

**Figure 3:**
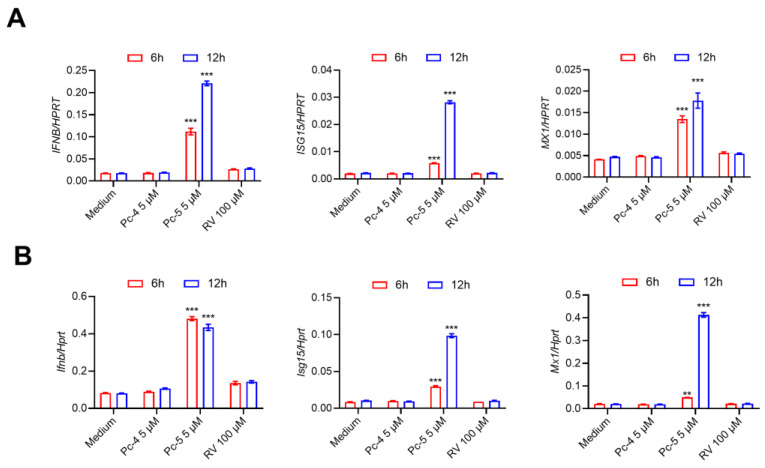
PC-5 upregulates the expression of interferon-beta and interferon-stimulated genes in vitro. (**A**) qPCR analysis of type I IFN-*IFNB* and its downstream genes *ISG15* and *MX1* in A549 cells at 6 h or 12 h with 5 µM cathelicidin Pc-4, Pc-5 and 100 µM RV. (**B**) qPCR analysis of peptides in mouse Raw264.7 cells after 6 h or 12 h. The expression levels of *Ifnb*, *Isg15* and *Mx1* are presented relative to those of the reference gene *Hprt* and the medium group. The data represent 3 independent experiments and are presented as the mean ± SEM. Statistical significance was determined by a two-way ANOVA. ** *p* < 0.01; *** *p* < 0.001.

The type I interferons and interferon-stimulated genes (ISGs) play multifaceted roles in the antiviral response, not only by directly inhibiting the viral replication and dissemination but also by modulating the function of immune cells and enhancing the overall immune response of the organism. In our study, we further investigated the effects of the antiviral peptides Pc-4 and Pc-5 on the expression of the type I interferons and ISGs. Interestingly, only the Pc-5 treatment of the A549 cells induced the production of IFNβ, ISG15, and MX1 (Figure 3A). The same effect was observed in mouse Raw264.7 cells (Figure 3B). These results suggest that the cathelicidin Pc-derived cyclic peptide 5 exerts antiviral activity by inducing IFN-β and ISGs, while Pc-4 may exert antiviral activity through other pathways.

### 2.4. The Cyclic Peptide Pc-5 Inhibits Viral Infections and Reduces Inflammatory Responses in Mice

**Figure 4:**
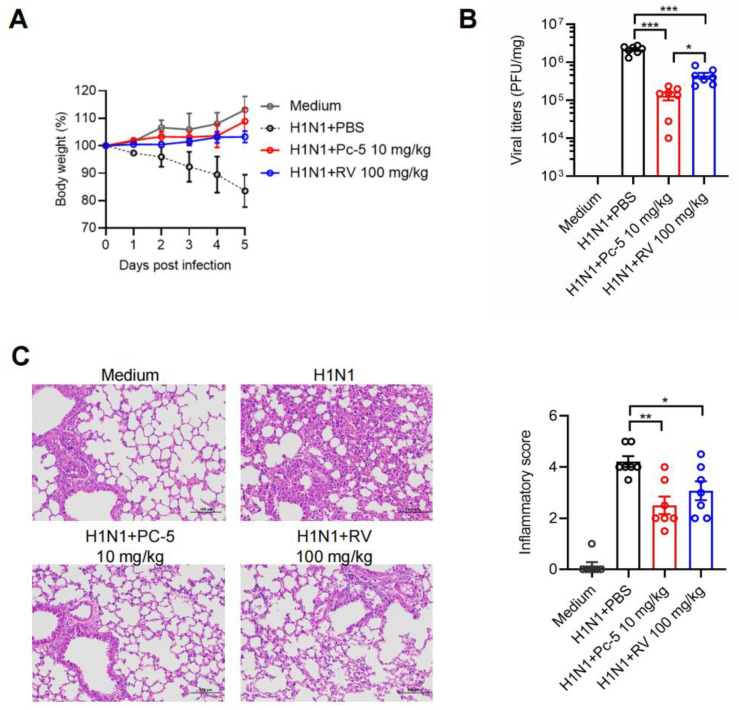
Pc-5 significantly restricts H1N1 infection in mice by reducing viral replication and associated pulmonary inflammation. (**A**) Following H1N1 infection, C57BL/6J mice were administered a daily intraperitoneal injection of either 10 mg/kg Pc-5 or 100 mg/kg RV or an equivalent volume of solvent as a control. The body weights of the mice were monitored over a period of five days postinfection (n = 7 mice). (**B**) Mean lung virus titers (n = 7 mice) on day 5 postinfection. (**C**) H&E staining was performed on lung sections. The scale bar represents 100 μm. The inflammatory score is shown as the mean ± SEM (n = 7 mice). Statistical significance was determined by a two-tailed Student’s *t*-test. * *p* < 0.01; ** *p* < 0.001; *** *p* < 0.001.

The advantages of cyclic peptides have increasingly positioned them as pivotal players in biomedical research and drug development. Their stability, penetrability, and innate bioactivity offer novel possibilities for treating a variety of diseases. In this context, we utilized mice infected with H1N1 to further assess the antiviral effects of cathelicidin Pc-5 in vivo. As depicted in Figure 4A, the treatment with 10 mg/kg Pc-5 or 100 mg/kg RV ameliorated the H1N1-induced impact on body growth. To further evaluate the antiviral efficiency of Pc-5 against H1N1, we analyzed the infectious viral titers in the pulmonary tissue of the mice. Notably, a significant reduction in the viral burden was observed on day 5 postinfection following treatment with Pc-5 and RV (Figure 4B). Moreover, the H1N1 influenza virus-infected lung tissue exhibited histological changes characterized by the formation of larger airspaces due to alveolar fusion and a marked increase in inflammatory infiltration between the tissues. However, with the addition of Pc-5 and RV, the pulmonary architecture reverted to a typical reticular pattern, and the lymphocytic infiltration correspondingly decreased. Compared with those in the H1N1 group, the inflammatory scores in the Pc-5 and RV treatment groups were lower (Figure 4C). Taken together, both Pc-5 and RV effectively inhibited H1N1 viral infection and the associated pulmonary inflammatory response. Furthermore, Pc-5 demonstrated superiority over RV in terms of dosage and therapeutic efficacy, highlighting its potential as a novel antiviral agent.

## 3. Discussion

Linear peptides derived from natural sources, such as animals, plants, marine life, amphibians, and others, are generally not optimal for direct therapeutic applications. This is due to several inherent limitations, including poor chemical and physical stability, a propensity for aggregation, a short half-life, inadequate absorption, suboptimal distribution, and the potential for multiple off-target interactions [28]. Nonetheless, the development and synthesis of innovative AVPs using natural AVPs as templates represents a highly effective strategy. This approach aims to mitigate the majority of the inherent limitations associated with natural AVPs while simultaneously augmenting their antiviral efficacy [11]. Building on this strategy, a peptide sequence designated Pc-1, derived from the cathelicidin of the pheasant and identified in the Antimicrobial Peptide Database 3, was optimized. The optimization process utilized Pc-1 as the original sequence, resulting in the formation of an amphipathic helix structure in the peptides. The optimization was carefully conducted to ensure that the sequences remained within a maximum length of 30 amino acids, concurrently preserving their functional α-helical domains. In a further refinement, charged amino acids were strategically incorporated to replace their uncharged counterparts to augment the antiviral potency. The ratio of polar to nonpolar residues was approximately 50%. Following modification, the net charge of the peptide was adjusted to +8, which was achieved by concentrating the positively charged amino acids. This concentration was intended to optimize the amphiphilic properties and enhance the antiviral efficacy of the peptide. As the optimization process continued, in the final stage of optimization, hydrophobic amino acids were strategically integrated into the hydrophilic face of the helical structure. This modification was designed to modulate the solubility of the peptides, thereby reducing the propensity for hemolytic reactions. As a result, a series of four antiviral peptides (AVPs) was designed. Among these, Pc-5 distinguished itself by adopting a cyclic polypeptide conformation due to the presence of cysteine (C) residues at its termini.

Influenza A viruses belong to the Orthomyxoviridae family of enveloped RNA viruses, whose earliest origins can be traced back to wild birds that usually migrate in autumn and carry the virus to different regions. Typically, the virus spreads through asymptomatic infections in these birds. However, on occasion, the virus is capable of crossing species barriers, leading to infections in a variety of hosts, including poultry, mammals, and humans [29]. Vaccine lag and drug resistance pose significant challenges in the fight against influenza virus infection. Given these limitations, there is an urgent unmet need for the development of novel antiviral drugs that are both safe and efficacious. In this context, antimicrobial peptides with antiviral activity have come to the forefront. Notably, the activity of the AVP Hylin-a1 secreted by the frog *Hypsiboas albopunctatus* has been reported against a variety of respiratory viruses, including the coronaviruses HCoV-229E and SARS-CoV-2, measles virus, human parainfluenza virus type 3, and influenza virus H1N1 [30]. The amphibian skin secretion ESC-1GN inhibits the entry of several H5N1 and H1N1 virus strains, with the IC50 values ranging from 1.29 to 4.59 μM [31]. In addition to other sources, avian species, notably pheasants, represent a significant reservoir for antiviral peptides, which have complex survival environments. Infection with the enveloped RNA virus infectious bronchitis virus (IBV) has been reported to upregulate the expression of antimicrobial peptides (avian β-defensins, including AvBD1, 2, 4–6, and cathelicidins, including CATH1 and CATH3) in avian species [32]. In this study, we discovered that Pc-1 and its derived peptides exhibited significant anti-H1N1 activity in vitro. Notably, Pc-4 at a half-maximal inhibitory concentration (1.81 ± 0.34 µM) and Pc-5 at a half-maximal inhibitory concentration (2.47 ± 1.95 μM) inhibited A549 cells. In comparison, the positive control drug ribavirin had a relatively weak effect on H1N1 replication, with a half-maximal effective inhibitory concentration of 101.30 ± 29.69 μM. Furthermore, cytotoxicity assays revealed an enhanced safety profile for the peptide following design modification. Type I interferon (IFN) is the host’s first line of defense against viral infection. In response to viral stimuli, cells produce and release the interferon, which binds to its receptor and initiates a signaling cascade response that results in the precise transcriptional regulation of hundreds of interferon-stimulated genes (ISGs). The expression products of the ISGs are integral to antiviral defense mechanisms, functioning either by directly impeding viral replication or by augmenting the cell’s immune surveillance capabilities, thereby facilitating the clearance of viral infections [33,34]. LL-37 not only inhibits viral replication with potent antiviral activity against enveloped VEEV but also regulates the expression of type I interferon (IFN) in infected cells [35]. LL-37 and CRAMP do not have direct virucidal effects on nonenveloped enterovirus EV71 but significantly modulate antiviral immune responses [36]. Our results demonstrated that the cyclic peptide Pc-5 substantially upregulated the expression of type I interferon and its downstream genes in both the A549 and Raw264.7 cells, and the expression levels were generally greater at 12 h than at 6 h. These findings indicate that the cyclic peptide Pc-5 intentionally augmented the host immune response, thereby exerting its antiviral effects. The Pc-5 inserted cysteines at both ends of the peptide chain based on the Pc-4, forming a cyclic structure. These cyclic polypeptides represent an innovative class of antiviral agents, characterized by enhanced pharmacological profiles. Therefore, we hypothesized that the cyclic structure of the Pc-5 enables it to trigger an intracellular signaling pathway, which in turn induces IFN production, while Pc-4 is unable to do so.

Compared with in vitro assays, in vivo testing more accurately reflects the clinical application of a drug. Therefore, it is both feasible and essential to evaluate the in vivo antiviral efficacy of the cyclic peptide Pc-5 using suitable mouse models. Severe acute influenza is known to induce an exaggerated neutrophil response, which can result in alveolar damage and a subsequent increase in the viral load [37]. Our study demonstrated that Pc-5 effectively reduced the viral load in the lungs and mitigated the progression of weight loss and inflammatory injury in H1N1-infected mice. These results suggest that Pc-5 may possess therapeutic potential when administered in vivo. Additionally, this research underscores the significance of harnessing host antimicrobial peptides in the development of novel antiviral therapeutics.

## 4. Materials and Methods

### 4.1. Cell Lines and Virus

Madin–Darby canine kidney (MDCK) cells and mouse mononuclear macrophage (RAW264.7) cells were cultured in DMEM (Gibco, Waltham, MA, USA), while the human lung adenocarcinoma (A549) cells were cultured in Dulbecco’s Modified Eagle’s Medium/Nutrient Mixture F-12 (DMEM/F-12) (Gibco, Waltham, MA, USA). In addition, they were supplemented with 10% (*v*/*v*) FBS (ExCell, Suzhou, China), 100 U/mL penicillin, and 100 µg/mL streptomycin in 5% CO_2_ at 37 °C.

The IAV strain A/Puerto Rico/8/1934 (PR8) was stored in our laboratory, and virus stocks were generated in the MDCK cells. Briefly, the confluent MDCK cells were inoculated for 1 h with the indicated H1N1 viruses at a multiplicity of infection (MOI) of 0.01. The cells were then washed and maintained in 1× MEM (0.3% BSA, 0.5 µg/mL N-tosyl-l-phenylalanyl chloromethyl ketone [TPCK]-treated trypsin) at 37 °C. After 48 h of incubation, the virus supernatants were collected and stored at −80 °C.

### 4.2. Peptide Design and Synthesis

We searched for the pheasant cathelicidin Pc-1 in the antimicrobial peptide database (http://aps.unmc.edu/AP/, accessed on 9 August 2023). Subsequently, sequence alignment was performed using the NCBI database to identify the complete sequence of the corresponding species, determine its sequence structure, and modify and synthesize the original sequence accordingly. The modifications were designed to adhere to the following criteria: the length of the modification should not exceed 30 amino acids; the net charge after modification should be eight; the charged amino acids should replace noncharged amino acids; the ratio of polar to nonpolar residues should be approximately 50%; the positively charged amino acids should be concentrated; and the hydrophobic amino acids should be embedded to reduce hemolytic reactions while preserving the α-helical functional region and enhancing the antiviral activity of the peptide. In accordance with the aforementioned principles, we designed the Pc-2, Pc-3, and Pc-4 peptides. Specifically, by inserting cysteine residues at positions 1 and 2 and between positions 14 and 15 in Pc-4, we engineered a cyclic peptide, designated Pc-5. The C-termini of all the peptides were amidated (-NH2). All the designed peptides were synthesized by GL Biochem (Shanghai) Ltd. (Shanghai, China) and analyzed by reverse-phase high-performance liquid chromatography (RP-HPLC) and mass spectrometry to confirm that their purity was greater than 95% (Tables S1 and S2).

### 4.3. CCK-8 Analysis

The A549 cells were cultured in 96-well plates and grown for 24 h, followed by H1N1 infection for 2 h and the addition of 0.0625, 1.25, 2.5 µM, 5 µM, 10 µM, 20 µM, and 40 µM of the designed peptides for 48 h. Then, we added a CCK-8 reagent, incubated the cells at 37 °C for 30 min, and detected the absorbance at 450 nm with a SpectraMax M2e Multi-Mode Microplate Reader (Molecular Devices, San Jose, CA, USA). To calculate the cell viability, the average OD values of the treated groups were compared to those of the untreated control group, which was considered 100% viable.

The formula for calculating cell viability is as follows:

Cell viability (%) = (average OD of treated sample/average OD of control sample) × 100%.

### 4.4. Plaque-Forming Assay

The cell supernatants collected from the cultures were serially diluted and subsequently added to the MDCK cells. The presence of the virus was determined by counting the plaques that formed. The viral titer was then calculated and expressed as plaque-forming units (PFUs) per milliliter (mL).

### 4.5. RNA Isolation and Real-Time PCR (qPCR)

The total RNA was extracted using a TRIzol reagent (Invitrogen, Waltham, MA, USA) according to the manufacturer’s protocol. The purity and concentration of the extracted RNA were assessed using a NanoDrop One UV-Vis spectrophotometer (Thermo Fisher Scientific, Waltham, MA, USA). The Evo M-MLV RT Kit (Accurate Biotechnology Co., Ltd., Changsha, China) was used according to the manufacturer’s instructions. One microgram of the RNA was reverse transcribed into cDNA. The diluted cDNAs were subjected to a qPCR analysis using ChamQ Universal SYBR qPCR Master Mix (Vazyme, Nanjing, China) with the following parameters: 95 °C for 30 s, followed by 40 cycles of 95 °C for 5 s and 60 °C for 30 s, followed by a melting curve analysis. All primers for the qPCR were synthesized by Sangon Biotech (Shanghai, China) (Table S3). The qPCR data were analyzed using the 2^−ΔΔCt^ method.

### 4.6. Animal Experiment

Female C57BL/6J mice, aged 6–8 weeks, were purchased from Beijing Vital River Laboratory Animal Technology Co., Ltd. (Beijing, China). After a 7-day acclimatization period, the mice were randomly divided into four groups, each comprising seven animals. The groups were as follows: a blank control group (PBS), a virus infection group, a Pc-1 treatment group for the H1N1 infection, and an RV treatment group for the H1N1 infection. The mice were infected with H1N1 via nasal inoculation at a titer of 1.5 × 10^6^ PFU per mouse. One hour postinfection, the mice in the Pc-5 and RV treatment groups received intraperitoneal injections of the Pc-5 (10 mg/kg) and RV (100 mg/kg), respectively, for 5 consecutive days [38,39]. The blank control group and the virus infection group received an equal volume of PBS as a control. Mouse body weight data were collected daily. Twenty-four hours after the final administration, the mice were euthanized for subsequent analysis.

We collected lung tissues for the viral titer analysis as described in the preceding methods. The viral titers are expressed as PFU per gram of the source tissue. Additionally, the lung tissue sections were prepared and fixed with 4% paraformaldehyde (PFA). The lung specimens were then embedded in paraffin, sectioned into 5 µm thick slices, and stained with hematoxylin and eosin (H&E). For the histological assessment of the lung tissue, images were evaluated in a blinded manner by researchers using a standardized scoring system as previously described [40].

### 4.7. Statistical Analysis

All the data presented were analyzed using GraphPad Prism software (version 8.0; GraphPad Software, Boston, MA, USA). The results are expressed as the mean ± SEM. A two-tailed Student’s *t*-test or a two-way ANOVA with multiple comparison correction was used to analyze the differences among the multiple groups. A *p *-value ≤ 0.05 indicated a statistically significant difference.

## Data Availability

The datasets used/analyzed during the current study are available from the corresponding author upon reasonable request.

## Supplementary Materials

The following supporting information can be downloaded at https://www.mdpi.com/article/10.3390/antibiotics13070606/s1: Table S1: The amino acid sequences of the designed peptides; Table S2: The physicochemical properties of the designed peptides; Table S3: The primer sequences.
