# Supplementary material for: A Cyclic Peptide Based on Pheasant Cathelicidin Inhibits Influenza A H1N1 Virus Infection
Source: Antibiotics (Basel). 2024 Jun 28;13(7):606. doi: 10.3390/antibiotics13070606 (PMC11273436; doi:10.3390/antibiotics13070606)
Supplement: Supplementary file 1 [file antibiotics-13-00606-s001.zip › antibiotics-3064362-supplementary.pdf]

Table S1. Amino acid sequences of the Designed Peptides.

Peptide	Sequence
Pc-1	RIKRFWPVVIRTVVAGYNLYRAIKKK-NH_2
Pc-2	KKWRKVIKKVVARYK-NH_2
Pc-3	FWKKVIKRVRKAVKK-NH_2
Pc-4	VVKKVRKLYKKIYKR-NH_2
Pc-5	VCVKKVRKLYKKIYKCR-NH_2

Table S2. Physicochemical properties of the Designed Peptides.

Peptide	Length	Molecular weight/Da	Theoretical pI	Netcharge	Instability index	Aliphatic index	*GRAVY*
Pc-1	26	3175.91	11.60	+8	13.26	112.31	−0.108
Pc-2	15	1930.46	11.30	+8	−12.67	90.67	−1.047
Pc-3	15	1914.46	12.05	+8	17.83	90.67	−0.773
Pc-4	15	1949.5	10.79	+8	12.17	110.00	−0.940
Pc-5	17	2155.78	10.24	+8	−11.70	97.06	−0.535

Table S3. Primer sequences.

Gene	Primer sequences（5'→3'）
Ifnb	GCCTTTGCCATCCAAGAGATGC<br>ACACTGTCTGCTGGTGGAGTTC
Mx1	GACCATAGGGGTCTTGACCAA<br>AGACTTGCTCTTTCTGAAAAGCC
Isg15	TGACTGTGAGAGCAAGCAGC<br>CCCCAGCATCTTCACCTTTA
Hprt	CTCATGGACTGATTATGGACAGGAC<br>GCAGGTCAGCAAAGAACTTATAGCC
IFNB	AGCTGAAGCAGTTCCAGAAG<br>AGTCTCATTCCAGCCAGTGC
MX1	GTTTCCGAAGTGGACATCGCA<br>CTGCACAGGTTGTTCTCAGC
ISG15	CGCAGATCACCCAGAAGATCG<br>TTCGTCGCATTTGTCCACCA
HPRT	GCTATAAATTCTTTGCTGACCTGCTG<br>AATTACTTTTATGTCCCCTGTTGACTGG
